# An Adverse Outcome Pathway Linking Organohalogen Exposure to Mitochondrial Disease

**DOI:** 10.1155/2019/9246495

**Published:** 2019-04-01

**Authors:** Brooke McMinn, Alicia L. Duval, Christie M. Sayes

**Affiliations:** Department of Environmental Science, Baylor University, One Bear Place # 97266, Waco, TX 76798-7266, USA

## Abstract

Adverse outcome pathways (AOPs) are pragmatic tools in human health hazard characterization and risk assessment. As such, one of the main goals of AOP development is to provide a clear, progressive, and linear mechanistic representation of pertinent toxicological key events (KEs) occurring along the different levels of biological organization. Here, we present an AOP framework that depicts how exposure to organohalogens can lead to mitochondrial disease. Organohalogens are disinfectant by-products (DBPs) found in our drinking water. Chloroform, trichloroacetic acid, and trichlorophenol were selected to represent specific types of organohalogens for the development of this AOP. Although each of these compounds contains chlorine atoms, they differ in aromaticity and solubility, which have a significant impact on their potency. This AOP consists of two main pathways, both of which are triggered by the molecular initiating event (MIE) of excessive reactive oxygen species generation. Pathway 1 details the downstream consequences of oxidative stress, which include mitochondrial DNA damage, protein aggregation, and depolarization of the mitochondrial membrane. Pathway 2 shows the KEs that result from inadequate supply of glutathione, including calcium dysregulation and ATP depletion. Pathways 1 and 2 converge at a common KE: opening of the mitochondrial membrane transition pore (mPTP). This leads to the release of cytochrome c, caspase activation, apoptosis, and mitochondrial disease. This AOP was developed according to the Organisation for Economic Co-operation and Development guidance, including critical consideration of the Bradford Hill criteria for Weight of Evidence assessment and key questions for evaluating confidence. The presented AOP is expected to serve as the basis for designing new toxicological tests as well as the characterization of novel biomarkers for disinfectant by-product exposure and adverse health effects.

## 1. Introduction

In the early 1960s, researchers identified mitochondrial disease as a serious clinical condition and have since increased their efforts to identify its etiology [1]. Mitochondrial diseases are progressive, chronic, and irreversible illnesses that result from failure of mitochondrial organelles, which are specialized cellular compartments responsible for generating more than 90% of the energy required to sustain life. In general, mitochondrial failure leads to cell injury, closely followed by cellular demise [2]. When multiple cells expire through this pathway, the most common adverse outcome (AO) is organ failure [3–6].

With the exception of red blood cells, all human cells contain mitochondria. Therefore, mitochondrial dysfunction can occur in nearly any organ system of the human body and cause a variety of adverse health conditions, ranging from mild (i.e., nausea or mild cognitive impairment) to severe (heart failure or Parkinson's Disease) [7–9]. The most common symptoms of mitochondrial dysfunction include loss of muscle coordination, muscle weakness, developmental delays, learning disabilities, heart disease, diabetes, gastrointestinal disorders, liver disease, kidney disease, and neurological problems [10–14].

Mitochondrial dysfunction becomes mitochondrial disease as soon as mutations caused by xenobiotic exposure, genetics, or a combination thereof are identified. Mutation markers can be found in either mitochondrial or nuclear DNA [15–17]. While most cases of mitochondrial disease are linked to a genetic malfunction, substantial evidence published in the recent literature suggests that environmental triggers or exposure to certain xenobiotics may be responsible for the onset of some mitochondrial diseases [18, 19]. Exposure to pesticides is one environmental trigger that is frequently cited in the literature [20–22]. Ingestion of disinfectant by-products (DBPs) from drinking water may also be a trigger of mitochondrial disease. Risk assessments performed on these environmental exposures provide evidence for addressing this growing public health concern [23–27].

Disinfectant products, such as chlorine or chloramine, are added to the drinking water supply to mitigate the onset of waterborne illnesses due to microorganisms. While this technology has greatly improved water quality around the world, the addition of chlorinated compounds to water has produced an unintended consequence: unnatural disinfectant by-products (DBPs) littering plumbing within the water distribution system (Table 1). Chlorine reacts readily with water constituents (such as metal ions and carbonaceous species) to produce DBPs, which have been associated with a variety of human health effects, including cancer [28–33]. A substantial amount of research has been performed investigating the chemical mechanisms of formation and subsequent compound identification [34–37]. However, fewer studies have reported the toxicological mechanisms of action after DBP exposure to humans [38–40]. The chemical mechanisms of DBP formation aid in exposure analyses, but, without adequate toxicological mechanisms of action reported, risk assessments are difficult to perform.

Toxicologists face many challenges when performing human risk assessments, such as incomplete dosimetry information, disparate* in vitro* and* in vivo* hazard results, and lack of human epidemiological data [41–43]. There is an ever-increasing number of substances (i.e. chemicals, particles, aerosols, pharmaceuticals, advanced materials, and by-products) that should be tested for toxicity, evaluated for exposure, and assessed for risk. Unfortunately, insufficient resources make these thorough analyses difficult to perform in a timely and cost-effective manner [44, 45]. Additionally, there is pressure to reduce the use of animals used in toxicological analyses [46]. Therefore, it is increasingly necessary to use available data from the literature to design, collect, and interpret new toxicological studies that answer unresolved questions or gaps in data [47, 48].

**Figure 1:**
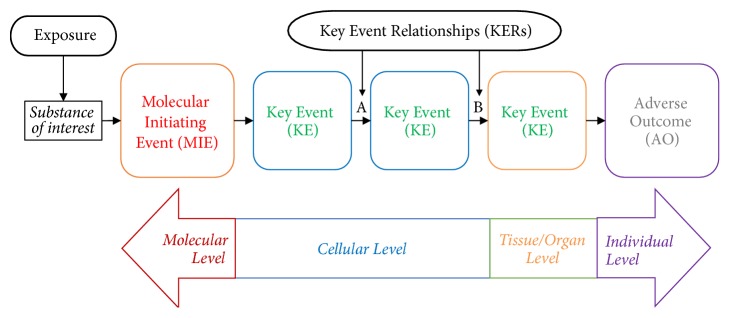
A schematic representation of the adverse outcome pathway (AOP). An AOP starts with a molecular initiating event (MIE) which is triggered by exposure to a substance of interest that interacts or reacts with a biological target, often a molecule. This MIE leads to a sequential series of intermediate key events (KE) along the different levels of biological organization to produce an adverse outcome (AO). A and B represent the relationships between two unique key events (KERs).

One promising pathway-based analysis tool is adverse outcome pathway (AOP) development (Figure 1). An AOP describes the progression of adverse health effects from lower-level molecular reactions to higher-level disease onset [47, 49]. AOP development begins with identifying a molecular initiating event (MIE) and ultimately concludes by recognizing the adverse outcome(s) (AO) of regulatory significance. The MIE and AO are related via a sequence of biologically plausible and scientifically supported key events (KEs) of increasing complexity. The relationships between the KEs are activated through structural and functional relationships coupled with weight of evidence criteria [50].

**Figure 2:**
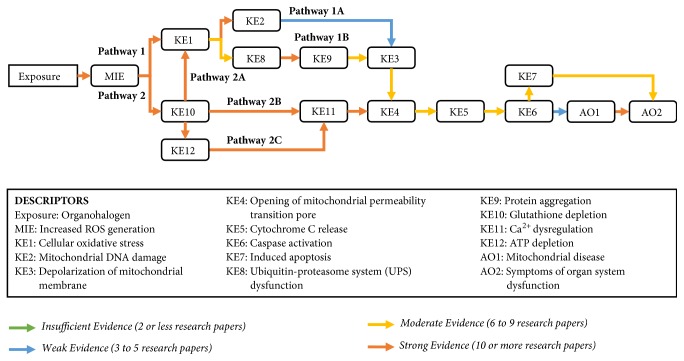
Weight of evidence assessment of key events (KE) and key event relationships supporting this AOP. All substances of interest (chloroform, chloroacetic acid, and chlorophenol) activate the proposed pathways.

The purpose of this manuscript is to explain and support a developed AOP that relates a global environmental exposure (ingestion of organohalogens) to mitochondrial dysfunction (opening of the mitochondrial permeability transition pore, mPTP) using individual mechanistic events identified in the peer-reviewed literature (Figure 2). This AOP has the potential to serve the scientific community as a basis for the development of new and targeted toxicological tests as well as the characterization of novel biomarkers of oxidative stress-induced organ system dysfunction.

## 2. Methods

### 2.1. AOP Development

There is no single strategy established that is suitable for all AOP development scenarios. Instead, AOP developers use different strategies to develop a single or network of pathways leading to adverse outcomes. The strategy most often utilized during the early stages of AOP development is data-mining [49]. This process includes analysis of relevant literature and database mining approaches to infer relationships between KEs (a.k.a, KERs). Mitochondrial disease AOPs are in their infancy; therefore, we used a combination of keyword searches. We conducted an extensive literature search using a combination of the following terms: “(mitochondrial*∗*) AND (AOP OR adverse outcome pathway)”. The primary databases searched were PubMed, Web of Science, and Scopus. The search results were not restricted by a date range; however, most papers included in this analysis were published within the last 20 years (1998-2018). A total of 70 unique papers were returned with these keyword phrases. On the other hand, papers linking “(mitochondrial*∗*) AND (environment*∗*)” are plentiful. A total of 15,221 unique papers were returned with these keywords. The next section describes the screening of these papers in an effort to narrow the developed AOP.

### 2.2. AOP Representation and Evaluation

The search terms “mitochondrial” AND “environmental” produced too much data to mine AOP key events and their associated relationships. From this initial search, we screened abstracts using specific terms related to different phases with in the AOP:

1. First, we included “organohalogen” and eliminated other environmental factors to build the molecular level.
2. Second, we included “mitochondria”, “oxidative stress”, and “glutathione” to build the cellular level.
3. Third, we included “mitochondrial permeability", “caspase activation”, “apoptosis” to develop the organ level. Articles that focused on mitochondrial dysfunction and genetic causes were excluded.

Upon applying inclusionary and exclusionary criteria, we were left with a total of 189 unique eligible articles. This process ensured that the articles with the most relevance were included in the final AOP.

**Figure 3:**
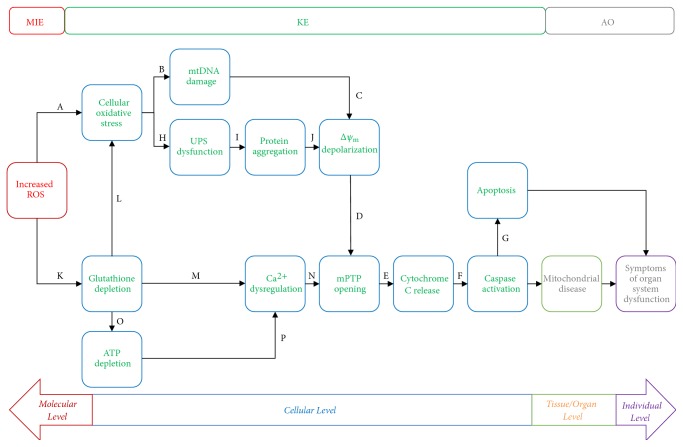
Graphical representation of organohalogen exposure to mitochondrial disease AOP. Key events at the molecular and cellular levels lead to AOs at the tissue/organ and individual levels. Exposure to organohalogens cause increased ROS production (Molecular Initiating Event, MIE), triggering the key events (KEs) that result in the adverse outcome (AO), defined as the onset of mitochondrial disease and the associated symptoms of organ symptom dysfunction. This AOP depicts 2 distinct pathways from MIE to AO.

**Figure 4:**
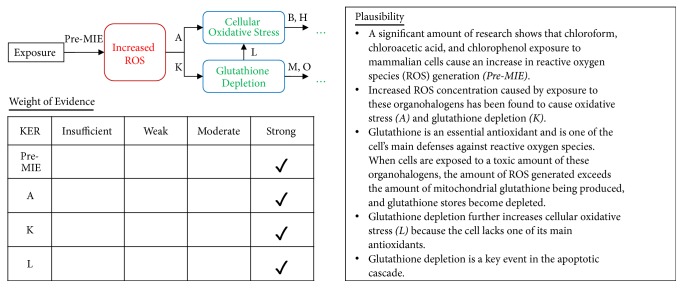
Exposure to MIE relationship is strong. A qualitative assessment of Key Event Relationships (KERs) in the AOP triggered by the generation of reactive oxygen species (MIE) and resulting in mitochondrial disease (AO) via KERs A, K, and L.

**Figure 5:**
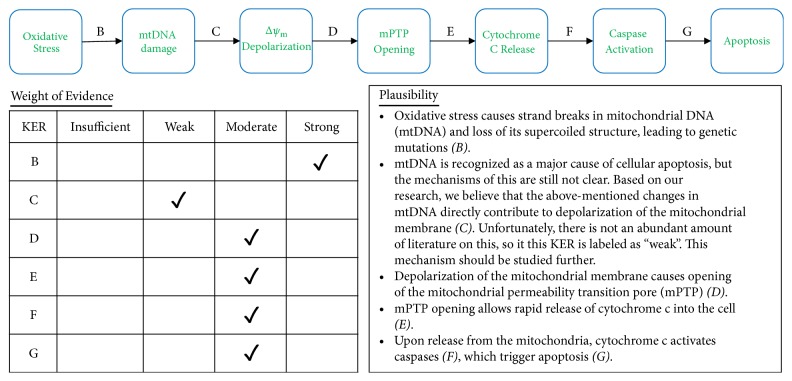
Analyses of Pathway IA. A qualitative assessments of Key Event Relationships (KERs) in the AOP triggered by the generation of reactive oxygen species (MIE) and resulting in mitochondrial disease (AO) via KERs B, C, D, E, F, and G.

**Figure 6:**
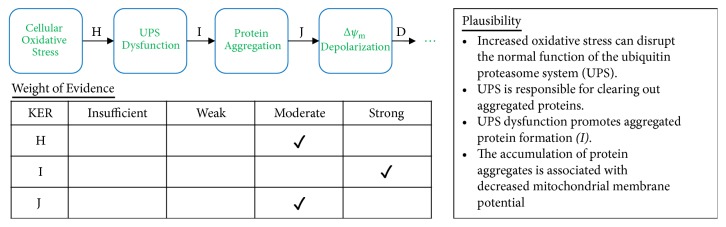
Analyses of Pathway IB. A qualitative assessments of Key Event Relationships (KERs) in the AOP triggered by the generation of reactive oxygen species (MIE) and resulting in mitochondrial disease (AO) via KERs H, I, and J.

**Figure 7:**
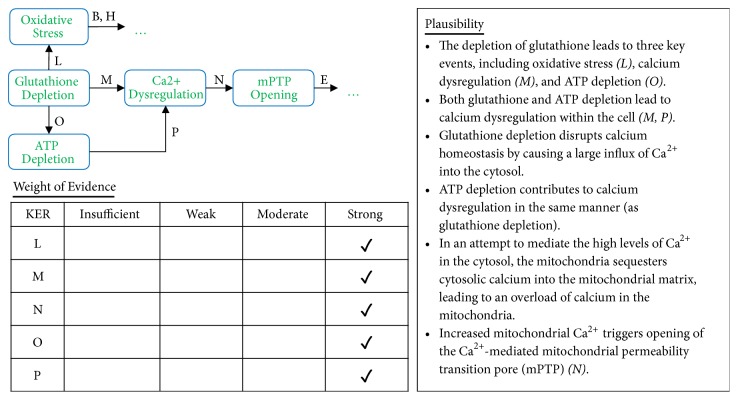
Analyses of Pathway II. A qualitative assessments of Key Event Relationships (KERs) in the AOP triggered by the generation of reactive oxygen species (MIE) and resulting in mitochondrial disease (AO) via KERs L, M, N, O, and P.

Appropriate KEs and their relationships were reported using a flow diagram showing the key event relationships (KERs) along the increasingly complicated levels of biological organization (i.e., molecular, cellular, tissue/organ, and individual levels) in a consecutive manner (Figure 1). Connections between events were decided based on a strength-based weight-of-evidence (WoE) assessment of the MIE, KE, and AO linkages (Figure 3). A final evaluation was conducted in two steps. First, Bradford Hill criteria were used to assess the WoE of the AOP to establish a causal link between the different blocks representing biological organization (Table 2). Second, we reported the confidence associated with each causal link by addressing OECD's proposed questions (Figures 4, 5, 6, and 7). This evaluation process ensures that the resultant AOP meets the minimal information requirements to establish plausibility of the proposed pathway.

## 3. Results

### 3.1. Weight of Evidence

#### 3.1.1. Empirical Support of the KERs

Based on well-established knowledge of mitochondrial function, the biological plausibility between increased ROS production and mitochondrial disease is strong. This is often associated with mitochondrial electron transport chain disruption or complex I inhibition [60–62]. Figure 2 contains the details for the biological plausibility of the KERs.

The degree of empirical support for the KERs ranges from* insufficient *to* strong* evidence. Organohalogens produce ROS species in water [63–67]. Many of these tri-chlorine containing compounds have a high affinity for disruption of mitochondrial electron transport chain (ETC) [63, 65]. Table 1 lists the physicochemical characteristics of three notable organohalogens (i.e., chloroform, trichloroacetic acid, and trichlorophenol) that are believed to inhibit the normal function of mitochondria in mammalian cells.

There is extensive indirect evidence of chloroform acting as an inhibitor of the electron transport chain through the generation of ROS, induction of oxidative stress conditions, and depletion of glutathione and ATP [68, 69]. Exposure to chloroform induces a dose-dependent cytotoxic response. It is classified as a Group 2B carcinogen by the IARC, however its propensity to induce tumors in humans is limited. LD_50_ values assessed from oral exposure to chloroform vary in rodent studies; values range 446 mg/kg for 14-day-olds male rats to 2,180 mg/kg for adult rats [51, 52]. But the mechanism of action at low-dose exposures is not yet established. Data suggest that chloroform metabolites produce DNA mutations [53, 54]. Chloroform-induced cell death is observed through the biochemical hallmarks of apoptosis, cytochrome c release, and activation of caspases 3 and 9. Cytochrome c is released into the cytosol in a time-dependent manner. The longer cells are exposed to organohalogens, the more cytochrome c is released.

Chloroacetic acid also interferes with the mitochondria. Unlike chloroform, it is not classified as a carcinogen. The metabolites of chloroacetic acid, including glycolic acid and oxalate, do generate ROS within the cytosol and quickly deplete glutathione supplies. The LD_50_ value of chloroacetic acid/trichloroacetic acid is reported to be 425 mg/kg (rats, oral) [55]. Chlorophenol acts in a similar manner but at lower doses (LD_50_ of 670 mg/kg (rats, oral)), making it more cytotoxic than chloroform (695 mg/kg), but less toxic than chloroacetic acid (425 mg/kg) [56–59]. Chloroacetic acid may be less cytotoxic because of its high-water solubility (greater than 10,000 g/L at 20°C). It is able to metabolize quickly through Phase 1 and Phase 2 processes as compared to the less water-soluble compounds (trichloromethane (8.09 g/L) and trichlorophenol (20.0 g/L)). Trichlorophenol may be more cytotoxic because of its aromatic structure. Aromatic compounds are generally metabolized by P450 enzymes and form epoxides, which cause DNA damage (mitochondrial or nuclear).

#### 3.1.2. Graphical Representation and Plausibility of the KERs

Many of the individual KERs within this developed AOP are strong. Figure 3 presents the overall graphical representation of organohalogen exposure to mitochondrial disease with each pathway's biological plausibility detailed in subsequent figures. Refer to Table 2 for the specific references from the scientific literature that support each of these KERs.

The electron transport chain, located on the inner membrane of the mitochondria, is the site of oxidative phosphorylation. When mammalian cells are exposed to organohalogens, their electron transport chain produces an increased amount of reactive oxygen species (ROS), inducing oxidative stress conditions within the cell (A). In an attempt to mediate oxidative stress, glutathione, a powerful antioxidant, is put to work. Eventually, ROS is produced faster than the cell produces antioxidants and glutathione stores are depleted (K), allowing the oxidative stress conditions to persist (L).

Because mitochondrial DNA is housed so close to the electron transport chain, it is exposed to high amounts of ROS, which causes strand breakage and loss of its tertiary structure (B). These changes affect mtDNA translation, eventually leading to depolarization of the mitochondrial membrane (C). Depolarization of the mitochondrial membrane contributes to opening of the mitochondrial permeability transition pore, or mPTP (D). When the mitochondrial membrane opens, cytochrome c is released into the cytosol of the cell (E). Cytochrome c activates caspases (F), which trigger the apoptotic cascade (G).

In addition to mitochondrial DNA damage, oxidative stress conditions also inhibit the function of the ubiquitin proteasome system (H), or UPS, which functions to clear out aggregated proteins within the cell. UPS dysfunction leads to a buildup of aggregated proteins within the cell (I). These aggregated proteins also contribute to depolarization of the mitochondrial membrane (J), which then triggers opening of the mPTP, cytochrome c release, caspase activation, and apoptosis.

Glutathione depletion caused by increased ROS production contributes to sustained oxidative stress conditions (L) and inhibits the production of ATP (O) in complex I of the mitochondrial electron transport chain. Both glutathione depletion and ATP depletion cause an interruption of calcium homeostasis within the cell (M, P). These conditions cause a large influx of calcium into the cell, which is then transported into the mitochondria in an attempt to regulate cellular calcium levels. This causes an overload of calcium in the mitochondria, which triggers opening of the calcium-mediated mPTP (N). Opening of the mPTP triggers cytochrome c release, caspase activation, and apoptosis, as discussed above.

Caspase activation causes two notable adverse biochemical reactions. The first is apoptosis, which occurs at the cellular level (G). The second is mitochondrial disease, which occurs at the tissue level. Apoptosis in cells with high mitochondrial content also contributes to the onset of mitochondrial disease. Mitochondria are responsible for providing cells with the energy needed to function normally. When mitochondrial disease is present, the cells are unable to function as efficiently, and therefore the organs are unable to function efficiently. This leads to symptoms of organ system failure or dysfunction. Apoptosis in cells with high mitochondria content can also lead to symptoms of organ dysfunction. Symptoms vary, depending on which organ(s) are affected by the mitochondrial dysfunction as well as which part of the organ is affected. For example, mitochondrial disease affecting the nigrostriatal pathway in the brain can cause motor deficits as manifested by Parkinson's disease [70]. Meanwhile, mitochondrial disease of the heart muscle can cause edema, shortness of breath, and arrhythmias, as manifested by mitochondrial cardiomyopathy [71].

It is important to note that many of the KEs presented in these pathways contain positive feedback loops. For example, calcium dysregulation, glutathione depletion, and ATP depletion all contribute to a further increase in ROS generation. These feedback loops were omitted from the figures for the sake of simplicity, but it is an important to recognize that these synergistic relationships do exist.

### 3.2. Applicability of the AOP

In terms of taxonomic applicability, this AOP describes the toxic mechanisms of organohalogens on mitochondrial electron transport chain and is therefore relevant to any organism that utilizes mitochondria for energy production. Mitochondrial disease can be caused by mitochondria respiratory deficiencies, which are often present at birth or in early childhood and typically result in progressive muscular and neurological degenerative disorders. Adult onset of mitochondria respiratory defects is increasingly common, thus increasing the applicability of organohalogens-to-mitochondrial disease AOP to multiple stages of life. Mitochondria serve several roles in maintaining homeostasis and these roles evolve from fertilized egg to old age. Although this AOP focuses on organohalogen exposure, this pathway can be adapted to fit a wide range of environmental triggers of mitochondrial disease.

## 4. Discussion

An AOP was created to best characterize the pathway-based analysis of organohalogen exposure that results in dysfunction of the mitochondrial electron transport chain in humans (Figure 1). For the purposes of this AOP, more emphasis was placed on the front-end of the AOP (MIE and the KEs) than the back-end (the AO). Mitochondrial disease can present itself in various physiological or toxicological manifestations, which makes it difficult to pinpoint one specific disease of interest. Instead, we provide evidence of the MIE responsible for initiating irreversible damage to mitochondria, which causes cell death and progression towards a diagnosable mitochondrial disease.

There is a suite of exogenous substances that have be shown to trigger changes in mitochondrial flux. While organohalogen compounds are highly cited as generators of ROS in cell and tissue systems, other materials, such as exhaust fumes, pesticides, tobacco products, smoke, drugs, or metals have been shown to increase oxidative stress in biological tests systems [72–74]. Furthermore, intangible stressors, such as radiation, heat, or ultraviolet light exposure, induce similar oxidative stress endpoints [75–78]. Most recently, exposure to engineered nanomaterials has been consistently linked to ROS generation in both cell-based and cell-free test systems which has been implicated as the main source of inflammatory responses in rodent studies [79–81].

Relating individual components of this AOP to other chemicals, particles, and fibers is possible. Inter-relationships between (or the ability to read-across) multiple test substances are dependent upon the level of biological organization. For example, the properties among classes of test substances vary greatly; i.e., chemicals are generally in a liquid form where particles and fibers are in a solid form. Liquids and solids have different physicochemical characteristics and hence the molecular initiating event is dependent upon the immediate chemical reaction between the substance of interest and subcellular entities; the physical (shape or size) and chemical (solubility or composition) characteristics often dictate the biochemical effects. Therefore, there are limitations in establishing interrelationships among different materials. However, as the AOP moves downstream from MIE to higher levels of biological organization, the dependency on physicochemical properties of the test system becomes less pronounced. Key events, key event relationships, and adverse outcomes at the tissue, organ, and individual levels are increasingly independent of material properties. Therefore, the ability to define interrelationships among classes of materials is possible.

The MIE is considered the “first domino” of the pathway, meaning that once the MIE occurs, reversible or irreversible damage will continuously cause KEs until the AO is achieved. Without the MIE, the progression of the AOP is halted and the KEs will not occur [47, 49, 50]. With regards to this AOP, the necessary MIE is the increase in ROS species. We have identified two main pathways that contribute to the development of mitochondrial disease from organohalogen exposure. These pathways diverge from the MIE and converge onto a single KE: opening of the mitochondrial permeability transition pore (mPTP).

Pathway 1 follows the direct effects of oxidative stress conditions caused by increased ROS generation. There are two main effects of oxidative stress identified in this pathway. The first is mitochondrial DNA (mtDNA) damage (Pathway 1A). Because ROS damages protein structures, the free radicals cause mtDNA strand breaks, loss of supercoiled structure, and interrupt mtDNA translation [82–84]. The second is dysfunction of the ubiquitin-proteasome system, which causes protein aggregation (Pathway 1B) [85, 86]. Both mtDNA damage and protein aggregation contribute to depolarization of the mitochondrial membrane, which in turn causes opening of the mPTP [87, 88].

Pathway 2 follows the direct effects of glutathione depletion caused by increased ROS concentration [89]. We have identified three effects of glutathione depletion. The first is that, because glutathione is one of the cell's main antioxidants, its depletion contributes to oxidative stress conditions (Pathway 2A) [90]. Glutathione depletion also causes a dysregulation in calcium homeostasis (Pathway 2B) [91]. When glutathione stores are depleted, a large amount of calcium is sent into the cell, which is then sequestered into the mitochondria and causes calcium overload [92]. This calcium overload within the mitochondria triggers opening of the Ca^2+^-mediated mPTP [93]. ROS generation and glutathione depletion perturb the mitochondrial electron transport chain [94]. As a result, the enzyme is unable to produce the proton gradient needed for ATP synthase to convert ADP to ATP, and ATP levels are depleted (Pathway 2C) [95]. ATP depletion contributes to calcium dysregulation in the same manner as glutathione depletion, as described above [96].

Pathways 1 and 2 converge on a common Key Event: opening of the mPTP. This is a crucial step in the apoptotic pathway, as it allows release of cytochrome c into the cytosol. Cytochrome c then activates caspases, which are responsible for programmed cell death.

The plausibility table included in Figure 5 outlines the research needed to strengthen the weight of evidence for key event C, i.e., mitochondrial DNA damage to depolarization of mitochondrial membrane. A few noteworthy studies have found evidence for this key event relationship [87, 97, 98]. Specifically, however, more evidence is needed to strengthen the KER by demonstrating that oxidative stress causes strand breaks in mitochondrial DNA (mtDNA) and subsequent loss of its supercoiled structure. Loss of structure is believed to lead to genetic mutations. Mitochondrial DNA (mtDNA) is recognized as a major cause of cellular apoptosis, but the exact mechanisms of toxic action are still not clear. Based on our research, we believe that the abovementioned changes in mtDNA directly contribute to depolarization of the mitochondrial membrane. Unfortunately, there is not an abundant amount of literature on event C so it this KER is labeled as “weak”. This mechanism should be studied further.

The Adverse Outcomes (AOs) identified in this AOP are mitochondrial disease (at the tissue/organ level) and symptoms of organ system dysfunction (at the individual level). The onset of mitochondrial disease occurs when enough cells in an organ exhibit significant caspase activation and/or induced apoptosis [13, 99]. Caspases cause programmed cell death via apoptosis [100]. After cell death, the tissue and organ to which the deceased cells belong become nonfunctional or nonviable, resulting in tissue and organ system failure. This systemic failure can lead to the typical clinical symptoms associated with mitochondrial disease, such as neurodegeneration, impaired growth, muscle weakness, and developmental delays, before evolving into a diagnosable mitochondrial disease (AO) [101, 102]. In other words, the inhibiting ability of organohalogen compounds has multi-level detrimental effects, including the macromolecular level, cellular/tissue level, organ level, organism level, and population or ecosystem level.

## 5. Conclusion

Mitochondrial function is an evolving field of study. Its role was previously described in simple terms, such as “mitochondria is the powerhouse of the cell”; however, new research is emerging that enables our understanding of mitochondria-to-disease pathways. With the motivation to reduce the number of animals in experimental designs and to create unprecedented value in the enormous amount of published studies on mitochondrial dysfunction, AOP development has the unique opportunity to serve as a framework for understanding mitochondrial disease. The effect of organohalogen exposure on intracellular respiration and oxidative stress conditions is one example in a long list of possible environmental exposures causing mitochondrial dysfunction. Overall, the biological plausibility of the key events and their relationships is strong and exists in the form of* in vivo* and* in vitro* studies. Although the strength of the biological plausibility of our AOP is strong, the strength of empirical evidence ranges from weak to strong, with the least amount of evidence existing to support the AO.

## Figures and Tables

**Table 1:** Organohalogen characterization table. The table includes physicochemical properties of three primary disinfectant by-products: chloroform, chloroacetic acid, and chlorophenol. The LD_50_ values of the materials after oral exposure to rats are also included. These organohalogens are known as mitochondrial toxins and potent disruptors of mitochondrial respiratory chain.

	Chloroform	Chloroacetic acid	Chlorophenol
IUPAC name	Trichloromethane	Trichloroacetic acid	2,4,6-Trichlorophenol

Chemical structure	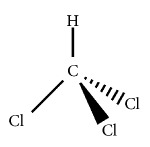	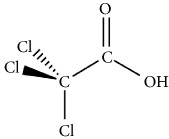	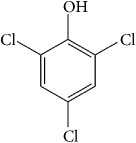

CAS number	67-66-3	76-03-9	88-06-2

Chemical formula	CHCl_3_	C_2_HCl_3_O_2_	C_6_H_3_Cl_3_O

Molecular weight	119.37 g/mol	163.38 g/mol	197.45 g/mol

Density	1.489 g/cm^3^ (20°C)	1.63 g/cm^3^ (20°C)	1.675 g/cm^3^ (20°C)

Water solubility	8.09 g/L (20°C)	>10,000 g/L (20°C)	20.0 g/L (20°C)

Dissociation (pKa)	15.7 (20°C)	0.66 (20°C)	8.56 (20°C)

Boiling point	61.2°C	195.5°C	246°C

Toxicity LD_50_ (rats,oal)	695 mg/kg [51–54]	425 mg/kg [55]	670 mg/kg [56–59]

**Table 2:** Organohalogen AOP References. The predominate pathways create a network of molecular events triggered during the development of mitochondrial disease. The key event relationships between each individual key event are presented here in tabular form.

Molecular Initiating Event	

Chloroform → increased ROS	(1) Chiu, C.-H., et al. Chloroform extract of solanum lyratum induced G0/G1 arrest via p21/p16 and induced apoptosis via reactive oxygen species, caspases and mitochondrial pathways in human oral cancer cell lines. *The American journal of Chinese medicine* 43.07 (2015): 1453. (2) Zhang, Y., et al. Chemical compositions and antiproliferation activities of the chloroform fraction from *P. fomentarius* in K562 cells. *Human & experimental toxicology* 34.7 (2015): 732. (3) Wang, Y., et al. Investigating migration inhibition and apoptotic effects of Fomitopsis pinicola chloroform extract on human colorectal cancer SW-480 cells. *PloS one* 9.7 (2014): e101303. (4) Looi, C.Y., et al. Induction of apoptosis in melanoma A375 cells by a chloroform fraction of *Centratherum anthelminticum (L.)* seeds involves NF-kappaB, p53 and Bcl-2-controlled mitochondrial signaling pathways. *BMC complementary and alternative medicine* 13.1 (2013): 166. (5) Faustino‐Rocha, A.I., et al. Trihalomethanes in liver pathology: Mitochondrial dysfunction and oxidative stress in the mouse. *Environmental toxicology* 31.8 (2016): 1009. (6) Ali, A., et al. Effect of drinking water disinfection by-products in human peripheral blood lymphocytes and sperm. *Mutation Research/Fundamental and Molecular Mechanisms of Mutagenesis* 770 (2014): 136. (7) Brunner, E.A., et al. Effects of anesthesia on intermediary metabolism. *Annual review of medicine* 26.1 (1975): 391.

Chloroform → decreased ATP	(1) Faustino‐Rocha, A. I., et al. Trihalomethanes in liver pathology: Mitochondrial dysfunction and oxidative stress in the mouse. *Environmental toxicology* 31.8 (2016): 1009. (2) Rottenberg, H. Uncoupling of oxidative phosphorylation in rat liver mitochondria by general anesthetics. *Proceedings of the National Academy of Sciences* 80.11 (1983): 3313. (3) Brunner, E.A., et al. Effects of anesthesia on intermediary metabolism. *Annual review of medicine* 26.1 (1975): 391.

Chloroform → decreased glutathione (GSH)	(1) Ekström, T., et al. Chloroform-induced glutathione depletion and toxicity in freshly isolated hepatocytes. *Biochemical pharmacology* 29.22 (1980): 3059. (2) Docks, E. L., et al. The role of glutathione in chloroform-induced hepatotoxicity. *Experimental and molecular pathology* 24.1 (1976): 13. (3) Beddowes, E.J., et al. Chloroform, carbon tetrachloride and glutathione depletion induce secondary genotoxicity in liver cells via oxidative stress. *Toxicology* 187.2-3 (2003): 101. (4) Wang, Y., et al. Investigating migration inhibition and apoptotic effects of Fomitopsis pinicola chloroform extract on human colorectal cancer SW-480 cells. *PloS one* 9.7 (2014): e101303. (5) Abbassi, R., et al. Chloroform-induced oxidative stress in rat liver: implication of metallothionein. *Toxicology and industrial health* 26.8 (2010): 487. (6) Hewitt, W.R., et al. Nephrotoxic interactions between ketonic solvents and halogenated aliphatic chemicals. *Toxicological Sciences* 4.6 (1984): 902. (7) Skrzypińska-Gawrysiak, M., et al. The hepatotoxic action of chloroform: short-time dynamics of biochemical alterations and dose-effect relationships. *Polish journal of occupational medicine and environmental health* 4.1 (1991): 77. (8) Azri-Meehan, S., et al. The hepatotoxicity of chloroform in precision-cut rat liver slices. *Toxicology* 73.3 (1992): 239. (9) Ekström, T., et al. Lipid peroxidation in vivo monitored as ethane exhalation and malondialdehyde excretion in urine after oral administration of chloroform. *Basic & Clinical Pharmacology & Toxicology* 58.4 (1986): 289. (10) Qin, L.-Q., et al. One-day dietary restriction changes hepatic metabolism and potentiates the hepatotoxicity of carbon tetrachloride and chloroform in rats. *The Tohoku journal of experimental medicine* 212.4 (2007): 379. (11) Cohen, P.J., et al. Continuous in vivo measurement of hepatic lipoperoxidation using chemiluminescence: halothane and chloroform compared. *Anesthesia and analgesia* 70.3 (1990): 296.

Chlorophenol → increased ROS	(1) Luo, Y., et al. 2-Chlorophenol induced hydroxyl radical production in mitochondria in Carassius auratus and oxidative stress–An electron paramagnetic resonance study. *Chemosphere* 71.7 (2008): 1260. (2) Luo, Y., et al. 2-Chlorophenol induced ROS generation in fish Carassius auratus based on the EPR method. *Chemosphere* 65.6 (2006): 1064. (3) Khachatryan, L., et al. Environmentally persistent free radicals (EPFRs). 1. Generation of reactive oxygen species in aqueous solutions. *Environmental science & technology* 45.19 (2011): 8559. (4) Atkinson, A., et al. Increased oxidative stress in the liver of mice treated with trichloroethylene. *Biochemistry and molecular biology international* 31.2 (1993): 297. (5) Igbinosa, E.O., et al. Toxicological profile of chlorophenols and their derivatives in the environment: the public health perspective. *The Scientific World Journal* (2013). (6) Bukowska, B., et al. Comparison of the effect of phenol and its derivatives on protein and free radical formation in human erythrocytes (in vitro). *Blood Cells, Molecules, and Diseases* 39.3 (2007): 238. (7) Michalowicz, J., et al. The Effects of 2, 4, 5-Trichlorophenol on Some Antioxidative Parameters and the Activity of Glutathione S-Transferase in Reed Canary Grass Leaves (Phalaris arudinacea). *Polish Journal of Environmental Studies* 18.5 (2009). (8) Li, Z., et al. Stress responses to trichlorophenol in Arabidopsis and integrative analysis of alteration in transcriptional profiling from microarray. *Gene* 555.2 (2015): 159. (9) Li, F., et al. Hydroxyl radical generation and oxidative stress in Carassius auratus liver as affected by 2, 4, 6-trichlorophenol. *Chemosphere* 67.1 (2007): 13. (10) Xia, Xi., et al. Response of selenium-dependent glutathione peroxidase in the freshwater bivalve Anodonta woodiana exposed to 2, 4-dichlorophenol, 2, 4, 6-trichlorophenol and pentachlorophenol. *Fish & shellfish immunology* 55 (2016): 499. (11) Dong, Y.-L., et al. Induction of oxidative stress and apoptosis by pentachlorophenol in primary cultures of Carassius carassius hepatocytes. *Comparative Biochemistry and Physiology Part C: Toxicology & Pharmacology* 150.2 (2009): 179.

Chlorophenol → decreased ATP	(1) Aschmann, C., et al. Short-term effects of chlorophenols on the function and viability of primary cultured rat hepatocytes. *Archives of toxicology* 63.2 (1989): 121. (2) Stockdale, M., et al. Effects of ring substituents on the activity of phenols as inhibitors and uncouplers of mitochondrial respiration. *The FEBS Journal* 21.4 (1971): 565. (3) Mitsuda, H., et al. Effect of chlorophenol analogues on the oxidative phosphorylation in rat liver mitochondria. *Agricultural and Biological Chemistry* 27.5 (1963): 366. (4) Stockdale, M., et al. Influence of ring substituents on the action of phenols on some dehydrogenases, phosphokinases and the soluble ATPase from mitochondria. *The FEBS Journal* 21.3 (1971): 416. (5) Hugül, M., et al. Modeling the kinetics of UV/hydrogen peroxide oxidation of some mono-, di-, and trichlorophenols. *Journal of hazardous materials* 77.1-3 (2000): 193. (6) Juhl, U., et al. The Induction of Dna Srtrand Breaks and Formation of Semiquinone Radicals by Metabolites of 2, 4, 5-Trichlorophenol. *Free radical research communications* 11.6 (1991): 295.

Chlorophenol → decreased glutathione	(1) Li, F., et al. Hydroxyl radical generation and oxidative stress in Carassius auratus liver as affected by 2, 4, 6-trichlorophenol. *Chemosphere* 67.1 (2007): 13. (2) Dong, Y.-L., et al. Induction of oxidative stress and apoptosis by pentachlorophenol in primary cultures of Carassius carassius hepatocytes. *Comparative Biochemistry and Physiology Part C: Toxicology & Pharmacology* 150.2 (2009): 179. (3) Wang, Y.-J., et al. Induction of glutathione depletion, p53 protein accumulation and cellular transformation by tetrachlorohydroquinone, a toxic metabolite of pentachlorophenol. *Chemico-biological interactions* 105.1 (1997): 1. (4) Valentovic, M., et al. 2-Amino-5-chlorophenol toxicity in renal cortical slices from Fischer 344 rats: effect of antioxidants and sulfhydryl agents. *Toxicology and applied pharmacology* 161.1 (1999): 1. (5) Luo, Y., et al. 2-Chlorophenol induced hydroxyl radical production in mitochondria in Carassius auratus and oxidative stress–An electron paramagnetic resonance study. *Chemosphere* 71.7 (2008): 1260. (6) Götz, R., et al. Effects of pentachlorophenol and 2, 4, 6-trichlorophenol on the disposition of sulfobromophthalein and respiration of isolated liver cells. *Archives of toxicology* 44.1-3 (1980): 147. (7) Ahammed, G.J., et al. 24-Epibrassinolide alleviates organic pollutants-retarded root elongation by promoting redox homeostasis and secondary metabolism in Cucumis sativus L. *Environmental Pollution* 229 (2017): 922.

Chloroacetic acid → increased ROS	(1) Lu, T.-H., et al. Chloroacetic acid triggers apoptosis in neuronal cells via a reactive oxygen species-induced endoplasmic reticulum stress signaling pathway. *Chemico-biological interactions* 225 (2015): 1. (2) Chen, C.-H., et al. Chloroacetic acid induced neuronal cells death through oxidative stress-mediated p38-MAPK activation pathway regulated mitochondria-dependent apoptotic signals. *Toxicology* 303 (2013): 72. (3) Pals, J., et al. Human cell toxicogenomic analysis linking reactive oxygen species to the toxicity of monohaloacetic acid drinking water disinfection byproducts. *Environmental science & technology* 47.21 (2013): 12514. (4) Pals, J., et al. Biological mechanism for the toxicity of haloacetic acid drinking water disinfection byproducts. *Environmental science & technology* 45.13 (2011): 5791. (5) Yin, J., et al. Comparative toxicity of chloro-and bromo-nitromethanes in mice based on a metabolomic method. *Chemosphere* 185 (2017): 20. (6) Ali, A., et al. Effect of drinking water disinfection by-products in human peripheral blood lymphocytes and sperm. *Mutation Research/Fundamental and Molecular Mechanisms of Mutagenesis* 770 (2014): 136. (7) Marsà, A., et al. Hazard assessment of three haloacetic acids, as byproducts of water disinfection, in human urothelial cells. *Toxicology and applied pharmacology* 347 (2018): 70. (8) Zhang, X., et al. 2, 4, 6-Trichlorophenol cytotoxicity involves oxidative stress, endoplasmic reticulum stress, and apoptosis. *International journal of toxicology* 33.6 (2014): 532. (9) Celik, I., et al. Hepatoprotective role and antioxidant capacity of pomegranate (Punica granatum) flowers infusion against trichloroacetic acid-exposed in rats. *Food and Chemical Toxicology* 47.1 (2009): 145. (10) Dad, A., et al. Pyruvate remediation of cell stress and genotoxicity induced by haloacetic acid drinking water disinfection by‐products. *Environmental and molecular mutagenesis* 54.8 (2013): 629. (11) Dad, A., et al. Haloacetic Acid Water Disinfection Byproducts Affect Pyruvate Dehydrogenase Activity and Disrupt Cellular Metabolism. *Environmental science & technology* 52.3 (2018): 1525.

Chloroacetic acid → decreased ATP	(1) Dad, A., et al. Pyruvate remediation of cell stress and genotoxicity induced by haloacetic acid drinking water disinfection by‐products. *Environmental and molecular mutagenesis* 54.8 (2013): 629. (2) Dad, A., et al. Haloacetic Acid Water Disinfection Byproducts Affect Pyruvate Dehydrogenase Activity and Disrupt Cellular Metabolism. *Environmental science & technology* 52.3 (2018): 1525. (3) Schmidt, M., et al. Effects of chlorinated acetates on the glutathione metabolism and on glycolysis of cultured astrocytes. *Neurotoxicity research* 19.4 (2011): 628.

Chloroacetic acid → decreased glutathione	(1) Chen, C.-H., et al. Chloroacetic acid induced neuronal cells death through oxidative stress-mediated p38-MAPK activation pathway regulated mitochondria-dependent apoptotic signals. *Toxicology* 303 (2013): 72. (2) Schmidt, M., et al. Effects of chlorinated acetates on the glutathione metabolism and on glycolysis of cultured astrocytes. *Neurotoxicity research* 19.4 (2011): 628. (3) Lu, T.-H., et al. Chloroacetic acid triggers apoptosis in neuronal cells via a reactive oxygen species-induced endoplasmic reticulum stress signaling pathway. *Chemico-biological interactions* 225 (2015): 1. (4) Bruschi, S., et al. In vitro cytotoxicity of mono-, di-, and trichloroacetate and its modulation by hepatic peroxisome proliferation. *Fundamental and Applied Toxicology* 21.3 (1993): 366.

Pathway 1A & Pathway 1B	

MIE → KE1 Increased ROS generation → oxidative stress	(1) Sun, F., et al. Environmental neurotoxic chemicals-induced ubiquitin proteasome system dysfunction in the pathogenesis and progression of Parkinson's disease. *Pharmacology & therapeutics* 114.3 (2007): 327. (2) Bender, A., et al. TOM40 mediates mitochondrial dysfunction induced by *α*-synuclein accumulation in Parkinson's disease. *PloS one* 8.4 (2013): e62277. (3) Hauser, D.N., et al. Mitochondrial dysfunction and oxidative stress in Parkinson's disease and monogenic parkinsonism. *Neurobiology of disease* 51 (2013): 35. (4) Mikhed, Y., et al. Mitochondrial oxidative stress, mitochondrial DNA damage and their role in age-related vascular dysfunction. *International journal of molecular sciences* 16.7 (2015): 15918. (5) Zhang, W., et al. Mediating effect of ROS on mtDNA damage and low ATP content induced by arsenic trioxide in mouse oocytes. *Toxicology in Vitro* 25.4 (2011): 979.

Pathway 1A	

KE1 → KE2 Oxidative stress causes mtDNA damage	(1) Bender, A., et al. TOM40 mediates mitochondrial dysfunction induced by *α*-synuclein accumulation in Parkinson's disease. *PloS one* 8.4 (2013): e62277. (2) Mikhed, Y., et al. Mitochondrial oxidative stress, mitochondrial DNA damage and their role in age-related vascular dysfunction. *International journal of molecular sciences* 16.7 (2015): 15918. (3) Zhang, W., et al. Mediating effect of ROS on mtDNA damage and low ATP content induced by arsenic trioxide in mouse oocytes. *Toxicology in Vitro* 25.4 (2011): 979. (4) Ayala-Peña, S. Role of oxidative DNA damage in mitochondrial dysfunction and Huntington's disease pathogenesis. *Free Radical Biology and Medicine* 62 (2013): 102. (5) Birch‐Machin, M.A., et al. Mitochondrial DNA damage as a biomarker for ultraviolet radiation exposure and oxidative stress. *British Journal of Dermatology* 169.s2 (2013): 9. (6) Santos, R.X., et al. Mitochondrial DNA oxidative damage and repair in aging and Alzheimer's disease. *Antioxidants & redox signaling* 18.18 (2013): 2444. (7) Yue, R., et al. Mitochondrial DNA oxidative damage contributes to cardiomyocyte ischemia/reperfusion‐injury in rats: cardioprotective role of lycopene. *Journal of cellular physiology* 230.9 (2015): 2128. (8) Han, Y., et al. Oxidative stress induces mitochondrial DNA damage and cytotoxicity through independent mechanisms in human cancer cells. *BioMed research international* 2013 (2013). (9) Chan, S.W., et al. Simultaneous quantification of mitochondrial DNA damage and copy number in circulating blood: a sensitive approach to systemic oxidative stress. *BioMed research international* 2013 (2013). (10) Wei, Y.-H. Mitochondrial DNA mutations and oxidative damage in aging and diseases: an emerging paradigm of gerontology and medicine. *Proceedings of the National Science Council, Republic of China. Part B, Life sciences* 22.2 (1998): 55. (11) Kim, Y.J., et al. Cytoplasmic ribosomal protein S3 (rpS3) plays a pivotal role in mitochondrial DNA damage surveillance. *Biochimica et Biophysica Acta (BBA)-Molecular Cell Research* 1833.12 (2013): 2943. (12) Basu, S., et al. Transcriptional mutagenesis by 8-oxodG in *α*-synuclein aggregation and the pathogenesis of Parkinson's disease. *Experimental & molecular medicine* 47.8 (2015): e179.

KE2→ KE3 mtDNA damage causes depolarization of mitochondrial membrane	(1) Kim, S.J., et al. The role of mitochondrial DNA in mediating alveolar epithelial cell apoptosis and pulmonary fibrosis. *International journal of molecular sciences* 16.9 (2015): 21486. (2) Santos, J.H., et al. Cell sorting experiments link persistent mitochondrial DNA damage with loss of mitochondrial membrane potential and apoptotic cell death. *Journal of biological chemistry* 278.3 (2003): 1728. (3) Ehlers, R.A., et al. Mitochondrial DNA damage and altered membrane potential (ΔΨ) in pancreatic acinar cells induced by reactive oxygen species. *Surgery* 126.2 (1999): 148.

KE3→KE4 Depolarization of mitochondrial membrane → opening of mPTP	(1) Bernardi, P. Modulation of the mitochondrial cyclosporin A-sensitive permeability transition pore by the proton electrochemical gradient. Evidence that the pore can be opened by membrane depolarization. *Journal of Biological Chemistry* 267.13 (1992): 8834. (2) Bernardi, P. Modulation of the mitochondrial cyclosporin A-sensitive permeability transition pore by the proton electrochemical gradient. Evidence that the pore can be opened by membrane depolarization. *Journal of Biological Chemistry* 267.13 (1992): 8834. (3) Petronilli, V., et al. Modulation of the mitochondrial cyclosporin A-sensitive permeability transition pore. II. The minimal requirements for pore induction underscore a key role for transmembrane electrical potential, matrix pH, and matrix Ca2+. *Journal of Biological Chemistry* 268.2 (1993): 1011. (4) Ly, J.D., et al. The mitochondrial membrane potential (Δ*ψ*m) in apoptosis; an update. *Apoptosis* 8.2 (2003): 115. (5) Scorrano, L., et al. On the voltage dependence of the mitochondrial permeability transition pore A critical appraisal. *Journal of Biological Chemistry* 272.19 (1997): 12295. (6) Petronilli, V., et al. The voltage sensor of the mitochondrial permeability transition pore is tuned by the oxidation-reduction state of vicinal thiols. Increase of the gating potential by oxidants and its reversal by reducing agents. *Journal of Biological Chemistry* 269.24 (1994): 16638.

KE4→KE5 Opening of mPTP → cytochrome c release	(1) Yang, B.-C., et al. Crotonaldehyde induces apoptosis in alveolar macrophages through intracellular calcium, mitochondria and p53 signaling pathways. *The Journal of toxicological sciences* 38.2 (2013): 225. (2) Yamamoto, T., et al. The mechanisms of the release of cytochrome C from mitochondria revealed by proteomics analysis. *Yakugaku zasshi: Journal of the Pharmaceutical Society of Japan* 132.10 (2012): 1099. (3) Tornero, D., et al. The role of the mitochondrial permeability transition pore in neurodegenerative processes. *Revista de neurologia* 35.4 (2002): 354. (4) Song, T., et al. Protection effect of atorvastatin in cerebral ischemia-reperfusion injury rats by blocking the mitochondrial permeability transition pore. *Genet Mol Res* 13.4 (2014): 10632. (5) Ma, X.D., et al. Mechanism of opening of mitochondrial permeability transition pore induced by arsenic trioxide. *Ai zheng Aizheng Chinese journal of cancer* 25.1 (2006): 17. (6) Crompton, Martin. The mitochondrial permeability transition pore and its role in cell death. *Biochemical Journal* 341.2 (1999): 233. (7) Ou, Z., et al. Mitochondrial-dependent mechanisms are involved in angiotensin II-induced apoptosis in dopaminergic neurons. *Journal of the renin-angiotensin-aldosterone system* 17.4 (2016): 1470320316672349. (8) Raza, H., et al. Acetylsalicylic acid-induced oxidative stress, cell cycle arrest, apoptosis and mitochondrial dysfunction in human hepatoma HepG2 cells. *European journal of pharmacology* 668.1-2 (2011): 15.

KE5→KE6 Cytochrome c release → activation of caspases	(1) Maria, D.A., et al. A novel proteasome inhibitor acting in mitochondrial dysfunction, ER stress and ROS production. *Investigational new drugs* 31.3 (2013): 493. (2) Spano, M., et al. The possible involvement of mitochondrial dysfunctions in Lewy body dementia: a systematic review. *Functional neurology* 30.3 (2015): 151. (3) Hashimoto, M., et al. Role of protein aggregation in mitochondrial dysfunction and neurodegeneration in Alzheimer's and Parkinson's diseases. *Neuromolecular medicine* 4.1-2 (2003): 21. (4) Kakimura, J.-I., et al. Release and aggregation of cytochrome c and *α*-synuclein are inhibited by the antiparkinsonian drugs, talipexole and pramipexole. *European journal of pharmacology* 417.1-2 (2001): 59. (5) Jiang, X., et al. Cytochrome C-mediated apoptosis. *Annual review of biochemistry* 73 (2004). (6) Garcia, M., et al. Mitochondria, motor neurons and aging. *Journal of the neurological sciences* 330.1 (2013): 18. (7) Lu, C., et al. Neuroprotective effects of tetramethylpyrazine against dopaminergic neuron injury in a rat model of Parkinson's disease induced by MPTP. *International journal of biological sciences* 10.4 (2014): 350. (8) Raza, H., et al. Acetylsalicylic acid-induced oxidative stress, cell cycle arrest, apoptosis and mitochondrial dysfunction in human hepatoma HepG2 cells. *European journal of pharmacology* 668.1-2 (2011): 15.

KE6→ KE7 Caspase activation causes apoptosis	(1) Hernandez-Baltazar, D., et al. Activation of GSK-3*β* and caspase-3 occurs in nigral dopamine neurons during the development of apoptosis activated by a striatal injection of 6-hydroxydopamine. *PloS one* 8.8 (2013): e70951. (2) Li, F., et al. Dysregulated expression of secretogranin III is involved in neurotoxin‐induced dopaminergic neuron apoptosis. *Journal of neuroscience research* 90.12 (2012): 2237. (3) Mei, J.-M., et al. Effects of CDNF on 6-OHDA-induced apoptosis in PC12 cells via modulation of Bcl-2/Bax and caspase-3 activation. *Neurological Sciences* 35.8 (2014): 1275. (4) Li, D.-W., et al. Guanosine exerts neuroprotective effects by reversing mitochondrial dysfunction in a cellular model of Parkinson's disease. *International journal of molecular medicine* 34.5 (2014): 1358. (5) Naoi, M., et al. Glutathione redox status in mitochondria and cytoplasm differentially and sequentially activates apoptosis cascade in dopamine-melanin-treated SH-SY5Y cells. *Neuroscience letters* 465.2 (2009): 118.

Pathway 1B	

KE1 → KE8 Oxidative stress causes UPS dysfunction	(1) Launay, N., et al. Oxidative stress regulates the ubiquitin–proteasome system and immunoproteasome functioning in a mouse model of X-adrenoleukodystrophy. *Brain* 136.3 (2013): 891. (2) Sun, F., et al. Environmental neurotoxic chemicals-induced ubiquitin proteasome system dysfunction in the pathogenesis and progression of Parkinson's disease. *Pharmacology & therapeutics* 114.3 (2007): 327. (3) Chondrogianni, N., et al. Protein damage, repair and proteolysis. *Molecular aspects of medicine* 35 (2014): 1. (4) Bendotti, C., et al. Dysfunction of constitutive and inducible ubiquitin-proteasome system in amyotrophic lateral sclerosis: implication for protein aggregation and immune response. *Progress in neurobiology* 97.2 (2012): 101. (5) Hauser, D.N., et al. Mitochondrial dysfunction and oxidative stress in Parkinson's disease and monogenic parkinsonism. *Neurobiology of disease* 51 (2013): 35. (6) Dias, V., et al. The role of oxidative stress in Parkinson's disease. *Journal of Parkinson's disease* 3.4 (2013): 461.

KE8→ KE9 UPS dysfunction causes protein aggregation	(1) Bendotti, C., et al. Dysfunction of constitutive and inducible ubiquitin-proteasome system in amyotrophic lateral sclerosis: implication for protein aggregation and immune response. *Progress in neurobiology* 97.2 (2012): 101. (2) Ebrahimi-Fakhari, D., et al. Protein degradation pathways in Parkinson's disease: curse or blessing. *Acta neuropathologica* 124.2 (2012): 153. (3) Riederer, B.M., et al. The role of the ubiquitin proteasome system in Alzheimer's disease. *Experimental Biology and Medicine* 236.3 (2011): 268. (4) Wu, J., et al. Effects of titanium dioxide nanoparticles on *α*-synuclein aggregation and the ubiquitin-proteasome system in dopaminergic neurons. *Artificial cells, nanomedicine, and biotechnology* 44.2 (2016): 690. (5) Chu, Y., et al. Alterations in lysosomal and proteasomal markers in Parkinson's disease: relationship to alpha-synuclein inclusions. *Neurobiology of disease* 35.3 (2009): 385. (6) Sass, M.B., et al. A pragmatic approach to biochemical systems theory applied to an *α*-synuclein-based model of Parkinson's disease. *Journal of neuroscience methods* 178.2 (2009): 366. (7) Sun, F., et al. Environmental neurotoxic chemicals-induced ubiquitin proteasome system dysfunction in the pathogenesis and progression of Parkinson's disease. *Pharmacology & therapeutics* 114.3 (2007): 327.

KE9→ KE3 Protein aggregation causes mitochondrial membrane depolarization	(1) Li, L., et al. Human A53T *α*-synuclein causes reversible deficits in mitochondrial function and dynamics in primary mouse cortical neurons. *PLoS One* 8.12 (2013): e85815. (2) Chen, M., et al. Age-dependent alpha-synuclein accumulation is correlated with elevation of mitochondrial TRPC3 in the brains of monkeys and mice. *Journal of Neural Transmission* 124.4 (2017): 441. (3) Luth, E.S., et al. Soluble, prefibrillar *α*-synuclein oligomers promote complex I-dependent, Ca2+-induced mitochondrial dysfunction. *Journal of Biological Chemistry* 289.31 (2014): 21490. (4) Sarafian, T.A., et al. Impairment of mitochondria in adult mouse brain overexpressing predominantly full-length, N-terminally acetylated human *α*-synuclein. *PloS one* 8.5 (2013): e63557. (5) He, Q., et al. Alpha-synuclein aggregation is involved in the toxicity induced by ferric iron to SK-N-SH neuroblastoma cells. *Journal of neural transmission* 118.3 (2011): 397. (6) Ebrahim, A.S., et al. Reduced expression of peroxisome-proliferator activated receptor gamma coactivator-1*α* enhances *α*-synuclein oligomerization and down regulates AKT/GSK3*β* signaling pathway in human neuronal cells that inducibly express *α*-synuclein. *Neuroscience letters* 473.2 (2010): 120. (7) Cleeter, M.W.J., et al. Glucocerebrosidase inhibition causes mitochondrial dysfunction and free radical damage. *Neurochemistry international* 62.1 (2013): 1.

Pathway 2	

MIE → KE10 Increased ROS causes glutathione depletion	(1) Mailloux, R., et al. Unearthing the secrets of mitochondrial ROS and glutathione in bioenergetics. *Trends in biochemical sciences* 38.12 (2013): 592. (2) Ross, E.K., et al. Immunocal® and preservation of glutathione as a novel neuroprotective strategy for degenerative disorders of the nervous system. *Recent patents on CNS drug discovery* 7.3 (2012): 230. (3) Meyer, A.J. The integration of glutathione homeostasis and redox signaling. *Journal of plant physiology* 165.13 (2008): 1390. (4) Hadi, T., et al. Glutathione prevents preterm parturition and fetal death by targeting macrophage-induced reactive oxygen species production in the myometrium. *The FASEB Journal* 29.6 (2015): 2653. (5) You, B.R., et al. Reactive oxygen species, glutathione, and thioredoxin influence suberoyl bishydroxamic acid-induced apoptosis in A549 lung cancer cells. *Tumor Biology* 36.5 (2015): 3429. (6) Timme-Laragy, A.R., et al. Glutathione redox dynamics and expression of glutathione-related genes in the developing embryo. *Free Radical Biology and Medicine* 65 (2013): 89. (7) You, B.R., et al. Gallic acid-induced lung cancer cell death is accompanied by ROS increase and glutathione depletion. *Molecular and cellular biochemistry* 357.1-2 (2011): 295. (8) Dunning, S., et al. Glutathione and antioxidant enzymes serve complementary roles in protecting activated hepatic stellate cells against hydrogen peroxide-induced cell death. *Biochimica et Biophysica Acta (BBA)-Molecular Basis of Disease* 1832.12 (2013): 2027. (9) You, B.R., et al. Arsenic trioxide induces human pulmonary fibroblast cell death via increasing ROS levels and GSH depletion. *Oncology reports* 28.2 (2012): 749. (10) Quintana-Cabrera, R., et al. Glutathione and *γ*-glutamylcysteine in the antioxidant and survival functions of mitochondria. (2013): 106. (11) Thushara, R.M., et al. Sesamol induces apoptosis in human platelets via reactive oxygen species-mediated mitochondrial damage. *Biochimie* 95.11 (2013): 2060.

Pathway 2A	

KE10 → KE1 Glutathione depletion causes oxidative stress	(1) Vaziri, N.D., et al. Induction of oxidative stress by glutathione depletion causes severe hypertension in normal rats. *Hypertension* 36.1 (2000): 142. (2) Schulz, J.B., et al. Glutathione, oxidative stress and neurodegeneration. *The FEBS Journal* 267.16 (2000): 4904. (3) Shang, Y., et al. Downregulation of glutathione biosynthesis contributes to oxidative stress and liver dysfunction in acute kidney injury. *Oxidative medicine and cellular longevity* 2016 (2016). (4) Trocino, R.A., et al. Significance of glutathione depletion and oxidative stress in early embryogenesis in glucose-induced rat embryo culture. *Diabetes* 44.8 (1995): 992. (5) Zlatković, J., et al. Chronic administration of fluoxetine or clozapine induces oxidative stress in rat liver: a histopathological study. *European Journal of Pharmaceutical Sciences* 59 (2014): 20. (6) Iguchi, Y., et al. Oxidative stress induced by glutathione depletion reproduces pathological modifications of TDP-43 linked to TDP-43 proteinopathies. *Neurobiology of disease* 45.3 (2012): 862. (7) Jung, C.L., et al. Synergistic activation of the Nrf2-signaling pathway by glyceollins under oxidative stress induced by glutathione depletion. *Journal of agricultural and food chemistry* 61.17 (2013): 4072. (8) Won, S.J., et al. Assessment at the single-cell level identifies neuronal glutathione depletion as both a cause and effect of ischemia-reperfusion oxidative stress. *Journal of Neuroscience* 35.18 (2015): 7143. (9) De Vos, C.H.R., et al. Glutathione depletion due to copper-induced phytochelatin synthesis causes oxidative stress in Silene cucubalus. *Plant physiology* 98.3 (1992): 853.

Pathway 2B	

KE10 →KE11 Glutathione depletion → Calcium dysregulation	(1) Övey, I.S., et al. Homocysteine and cytosolic GSH depletion induce apoptosis and oxidative toxicity through cytosolic calcium overload in the hippocampus of aged mice: involvement of TRPM2 and TRPV1 channels. *Neuroscience* 284 (2015): 225. (2) Frosali, S., et al. Role of intracellular calcium and S-glutathionylation in cell death induced by a mixture of isothiazolinones in HL60 cells. *Biochimica et Biophysica Acta (BBA)-Molecular Cell Research* 1793.3 (2009): 572. (3) Orihuela, D., et al. Aluminium-induced impairment of transcellular calcium absorption in the small intestine: calcium uptake and glutathione influence. *Journal of inorganic biochemistry* 99.9 (2005): 1879. (4) Macho, A., et al. Glutathione depletion is an early and calcium elevation is a late event of thymocyte apoptosis. *The Journal of Immunology* 158.10 (1997): 4612. (5) Grewal, K.K., et al. Bromobenzene and furosemide hepatotoxicity: alterations in glutathione, protein thiols, and calcium. *Canadian journal of physiology and pharmacology* 74.3 (1996): 257. (6) Singh, B.K., et al. Nimesulide aggravates redox imbalance and calcium dependent mitochondrial permeability transition leading to dysfunction in vitro. *Toxicology* 275.1-3 (2010): 1. (7) Vendemiale, G., et al. Effect of acetaminophen administration on hepatic glutathione compartmentation and mitochondrial energy metabolism in the rat. *Biochemical pharmacology* 52.8 (1996): 1147. (8) Marchionatti, A.M., et al. Mitochondrial dysfunction is responsible for the intestinal calcium absorption inhibition induced by menadione. *Biochimica et Biophysica Acta (BBA)-General Subjects* 1780.2 (2008): 101. (9) Özgül, C., et al. TRPM2 channel protective properties of N-acetylcysteine on cytosolic glutathione depletion dependent oxidative stress and Ca2+ influx in rat dorsal root ganglion. *Physiology & behavior* 106.2 (2012): 122. (10) Yang, B.-C., et al. Crotonaldehyde induces apoptosis in alveolar macrophages through intracellular calcium, mitochondria and p53 signaling pathways. *The Journal of toxicological sciences* 38.2 (2013): 225. (11) Övey, I.S., et al. Homocysteine and cytosolic GSH depletion induce apoptosis and oxidative toxicity through cytosolic calcium overload in the hippocampus of aged mice: involvement of TRPM2 and TRPV1 channels. *Neuroscience* 284 (2015): 225. (12) Thushara, R.M., et al. Sesamol induces apoptosis in human platelets via reactive oxygen species-mediated mitochondrial damage. *Biochimie* 95.11 (2013): 2060. (13) Nazıroğlu, M., et al. Neuroprotection induced by N-acetylcysteine against cytosolic glutathione depletion-induced Ca2+ influx in dorsal root ganglion neurons of mice: role of TRPV1 channels. *Neuroscience* 242 (2013): 151.

KE11→ KE4 Calcium dysregulation causes opening of mPTP	(1) Lu, C., et al. Role of calcium and cyclophilin D in the regulation of mitochondrial permeabilization induced by glutathione depletion. *Biochemical and biophysical research communications* 363.3 (2007): 572. (2) Baumgartner, H.K., et al. Calcium elevation in mitochondria is the main Ca2+ requirement for mitochondrial permeability transition pore (mPTP) opening. *Journal of Biological Chemistry* 284.31 (2009): 20796. (3) Thushara, R.M., et al. Sesamol induces apoptosis in human platelets via reactive oxygen species-mediated mitochondrial damage. *Biochimie* 95.11 (2013): 2060. (4) Zhang, S., et al. Critical roles of intracellular thiols and calcium in parthenolide-induced apoptosis in human colorectal cancer cells. *Cancer letters* 208.2 (2004): 143. (5) Bernardi, P. Mitochondria in muscle cell death. *The Italian Journal of Neurological Sciences* 20.6 (1999): 395. (6) Yang, B.-C., et al. Crotonaldehyde induces apoptosis in alveolar macrophages through intracellular calcium, mitochondria and p53 signaling pathways. *The Journal of toxicological sciences* 38.2 (2013): 225. (7) Berson, A., et al. The anti-inflammatory drug, nimesulide (4-nitro-2-phenoxymethane-sulfoanilide), uncouples mitochondria and induces mitochondrial permeability transition in human hepatoma cells: protection by albumin. *Journal of Pharmacology and Experimental Therapeutics* 318.1 (2006): 444. (8) Lee, G.-H., et al. Bax inhibitor-1-mediated inhibition of mitochondrial Ca 2+ intake regulates mitochondrial permeability transition pore opening and cell death. *Scientific reports* 4 (2014): 5194. (9) Barsukova, A., et al. Activation of the mitochondrial permeability transition pore modulates Ca2+ responses to physiological stimuli in adult neurons. *European Journal of Neuroscience* 33.5 (2011): 831. (10) Moon, S.H., et al. Genetic ablation of calcium-independent phospholipase A2*γ* (iPLA2*γ*) attenuates calcium-induced opening of the mitochondrial permeability transition pore and resultant cytochrome c release. *Journal of Biological Chemistry* 287.35 (2012): 29837. (11) Yamamoto, T., et al. The mechanisms of the release of cytochrome C from mitochondria revealed by proteomics analysis. *Yakugaku zasshi: Journal of the Pharmaceutical Society of Japan* 132.10 (2012): 1099. (12) Rasola, A., et al. The mitochondrial permeability transition pore and its involvement in cell death and in disease pathogenesis. *Apoptosis* 12.5 (2007): 815. (13) De la Fuente, S., et al. The Spatial Distribution of the Na+/Ca2+ Exchanger in Cardiac Mitochondria Enhances the Efficincy of the Mitochondrial Ca2+ Signal Generation. *Biophysical Journal* 114.3 (2018): 659a. (14) Kannurpatti, S.S., et al. Calcium sequestering ability of mitochondria modulates influx of calcium through glutamate receptor channel. *Neurochemical research* 25.12 (2000): 1527. (15) Rueda, C.B., et al. Glutamate excitotoxicity and Ca2+-regulation of respiration: Role of the Ca2+ activated mitochondrial transporters (CaMCs). *Biochimica et Biophysica Acta (BBA)-Bioenergetics* 1857.8 (2016): 1158. (16) Baines, C.P., et al. Loss of cyclophilin D reveals a critical role for mitochondrial permeability transition in cell death. *Nature* 434.7033 (2005): 658. (17) Basso, E., et al. Properties of the permeability transition pore in mitochondria devoid of Cyclophilin D. *Journal of Biological Chemistry* 280.19 (2005): 18558.

Pathway 2C	

KE10→ KE12 Glutathione depletion causes ATP depletion	(1) Almeida, A., et al. Glutamate neurotoxicity is associated with nitric oxide-mediated mitochondrial dysfunction and glutathione depletion. *Brain research* 790.1-2 (1998): 209. (2) Vesce, S., et al. Acute glutathione depletion restricts mitochondrial ATP export in cerebellar granule neurons. *Journal of Biological Chemistry* 280.46 (2005): 38720. (3) Schütt, F., et al. Moderately reduced ATP levels promote oxidative stress and debilitate autophagic and phagocytic capacities in human RPE cells. *Investigative ophthalmology & visual science* 53.9 (2012): 5354. (4) Huang, J., et al. Cellular responses of cultured cerebellar astrocytes to ethacrynic acid-induced perturbation of subcellular glutathione homeostasis. *Brain research* 711.1-2 (1996): 184. (5) Zeevalk, G.D., et al. Energy Stress‐Induced Dopamine Loss in Glutathione Peroxidase‐Overexpressing Transgenic Mice and in Glutathione‐Depleted Mesencephalic Cultures. *Journal of neurochemistry* 68.1 (1997): 426. (6) Den Boer, P.J., et al. Effect of glutathione depletion on the cytotoxicity of xenobiotics and induction of single-strand DNA breaks by ionizing radiation in isolated hamster round spermatids. *Journal of reproduction and fertility* 88.1 (1990): 259. (7) Vesce, S., et al. Acute glutathione depletion restricts mitochondrial ATP export in cerebellar granule neurons. *Journal of Biological Chemistry* 280.46 (2005): 38720. (8) Navarini, A.L.F., et al. Hydroxychalcones induce apoptosis in B16-F10 melanoma cells via GSH and ATP depletion. *European journal of medicinal chemistry* 44.4 (2009): 1630. (9) Locatelli, C., et al. Gallic acid ester derivatives induce apoptosis and cell adhesion inhibition in melanoma cells: the relationship between free radical generation, glutathione depletion and cell death. *Chemico-biological interactions* 181.2 (2009): 175. (10) Raza, H., et al. Acetylsalicylic acid-induced oxidative stress, cell cycle arrest, apoptosis and mitochondrial dysfunction in human hepatoma HepG2 cells. *European journal of pharmacology* 668.1-2 (2011): 15. (11) Mithöfer, K., et al. Mitochondrial poisons cause depletion of reduced glutathione in isolated hepatocytes. *Archives of biochemistry and biophysics* 295.1 (1992): 132.

KE12→ KE11 ATP depletion causes calcium dysregulation	(1) Bruce, J.I.E. Plasma membrane calcium pump regulation by metabolic stress. *World journal of biological chemistry* 1.7 (2010): 221. (2) Spivey, J.R., et al. Glycochenodeoxycholate-induced lethal hepatocellular injury in rat hepatocytes. Role of ATP depletion and cytosolic free calcium. *The Journal of clinical investigation* 92.1 (1993): 17. (3) Akopova, O.V., et al. The effect of ATP-dependent K (+)-channel opener on the functional state and the opening of cyclosporine-sensitive pore in rat liver mitochondria. *Ukrains' kyi biokhimichnyi zhurnal (1999)* 85.3 (2013): 38. (4) Zhang, R., et al. Involvement of calcium, reactive oxygen species, and ATP in hexavalent chromium-induced damage in red blood cells. *Cellular Physiology and Biochemistry* 34.5 (2014): 1780. (5) James, A.D., et al. Glycolytic ATP fuels the plasma membrane calcium pump critical for pancreatic cancer cell survival. *Journal of Biological Chemistry* 288.50 (2013): 36007. (6) James, A.D., et al. The plasma membrane calcium pump in pancreatic cancer cells exhibiting the Warburg effect relies on glycolytic ATP. *Journal of Biological Chemistry* 290.41 (2015): 24760. (7) Cejka, J.-C., et al. Activation of calcium influx by ATP and store depletion in primary cultures of renal proximal cells. *Pflügers Archiv* 427.1-2 (1994): 33. (8) Chao, C.‐C., et al. Ca2+ store depletion and endoplasmic reticulum stress are involved in P2X7 receptor‐mediated neurotoxicity in differentiated NG108‐15 cells. *Journal of cellular biochemistry* 113.4 (2012): 1377. (9) Mattson, M.P., et al. Energetics and oxidative stress in synaptic plasticity and neurodegenerative disorders. *Neuromolecular medicine* 2.2 (2002): 215. (10) Bernardi, P. Mitochondria in muscle cell death. *The Italian Journal of Neurological Sciences* 20.6 (1999): 395. (11) Berson, A., et al. The anti-inflammatory drug, nimesulide (4-nitro-2-phenoxymethane-sulfoanilide), uncouples mitochondria and induces mitochondrial permeability transition in human hepatoma cells: protection by albumin. *Journal of Pharmacology and Experimental Therapeutics* 318.1 (2006): 444.

Adverse Outcomes	

KE7→ AO1 Apoptosis causes mitochondrial disease	(1) Du, Ya., et al. Minocycline prevents nigrostriatal dopaminergic neurodegeneration in the MPTP model of Parkinson's disease. *Proceedings of the National Academy of Sciences* 98.25 (2001): 14669. (2) Chung, K.K.K., et al. New insights into Parkinson's disease. *Journal of neurology* 250.3 (2003): iii15. (3) Thomas, K.J., et al. The role of PTEN-induced kinase 1 in mitochondrial dysfunction and dynamics. *The international journal of biochemistry & cell biology* 41.10 (2009): 2025. (4) Nordström, U., et al. Progressive nigrostriatal terminal dysfunction and degeneration in the engrailed1 heterozygous mouse model of Parkinson's disease. *Neurobiology of disease* 73 (2015): 70. (5) Van der Perren, A., et al. Longitudinal follow-up and characterization of a robust rat model for Parkinson's disease based on overexpression of alpha-synuclein with adeno-associated viral vectors. *Neurobiology of aging* 36.3 (2015): 1543. (6) Ares-Santos, S., et al. Methamphetamine causes degeneration of dopamine cell bodies and terminals of the nigrostriatal pathway evidenced by silver staining. *Neuropsychopharmacology* 39.5 (2014): 1066. (7) Gantz, S.C., et al. Loss of Mecp2 in substantia nigra dopamine neurons compromises the nigrostriatal pathway. *Journal of Neuroscience* 31.35 (2011): 12629. (8) Panayotis, N., et al. Morphological and functional alterations in the substantia nigra pars compacta of the Mecp2-null mouse. *Neurobiology of disease* 41.2 (2011): 385. (9) Matsuura, K., et al. Cyclosporin A attenuates degeneration of dopaminergic neurons induced by 6-hydroxydopamine in the mouse brain. *Brain research* 733.1 (1996): 101. (10) Walters, T.L., et al. Diethyldithiocarbamate causes nigral cell loss and dopamine depletion with nontoxic doses of MPTP. *Experimental neurology* 156.1 (1999): 62. (11) Raza, H., et al. Acetylsalicylic acid-induced oxidative stress, cell cycle arrest, apoptosis and mitochondrial dysfunction in human hepatoma HepG2 cells. *European journal of pharmacology* 668.1-2 (2011): 15.

AO1 → AO2 Mitochondrial disease causes symptoms of organ system failure	(1) Crouser, E.D. Mitochondrial dysfunction in septic shock and multiple organ dysfunction syndrome. *Mitochondrion* 4.5 (2004): 729. (2) Ares-Santos, S., et al. Methamphetamine causes degeneration of dopamine cell bodies and terminals of the nigrostriatal pathway evidenced by silver staining. *Neuropsychopharmacology* 39.5 (2014): 1066. (3) Colebrooke, R.E., et al. Age‐related decline in striatal dopamine content and motor performance occurs in the absence of nigral cell loss in a genetic mouse model of Parkinson's disease. *European Journal of Neuroscience* 24.9 (2006): 2622. (4) Panayotis, N., et al. Morphological and functional alterations in the substantia nigra pars compacta of the Mecp2-null mouse. *Neurobiology of disease* 41.2 (2011): 385. (5) Willard, A.M., et al. Differential degradation of motor deficits during gradual dopamine depletion with 6-hydroxydopamine in mice. *Neuroscience* 301 (2015): 254. (6) Heuer, A., et al. Dopamine-rich grafts alleviate deficits in contralateral response space induced by extensive dopamine depletion in rats. *Experimental neurology* 247 (2013): 485. (7) Plowman, E.K., et al. Differential sensitivity of cranial and limb motor function to nigrostriatal dopamine depletion. *Behavioural brain research* 237 (2013): 157. (8) Plowman, E.K., et al. Striatal dopamine depletion induces forelimb motor impairments and disrupts forelimb movement representations within the motor cortex. *Journal of Parkinson's disease* 1.1 (2011): 93. (9) Christopher, L., et al. Combined insular and striatal dopamine dysfunction are associated with executive deficits in Parkinson's disease with mild cognitive impairment. *Brain* 137.2 (2013): 565. (10) Plowman, E.K., et al. Behavioral and neurophysiological correlates of striatal dopamine depletion: A rodent model of Parkinson's disease. *Journal of communication disorders* 44.5 (2011): 549. (11) Charlton, C.G., et al. Striatal dopamine depletion, tremors, and hypokinesia following the intracranial injection of S-adenosylmethionine. *Molecular and chemical neuropathology* 26.3 (1995): 269.

## Data Availability

The data used to support the findings of this study are available from the corresponding author upon request.

## References

[1] Scaglia F. (2004). Clinical spectrum, morbidity, and mortality in 113 pediatric patients with mitochondrial disease. *Pediatrics*.

[2] Tzoulis C., Schwarzlmüller T., Biermann M., Haugarvoll K., Bindoff L. A. (2016). Mitochondrial DNA homeostasis is essential for nigrostriatal integrity. *Mitochondrion*.

[3] Korsmeyer S. J. (1999). BCL-2 gene family and the regulation of programmed cell death. *Cancer Genetics and Cytogenetics*.

[4] Kroemer G., Reed J. C. (2000). Mitochondrial control of cell death. *Nature Medicine*.

[5] Linkermann A., Stockwell B. R., Krautwald S., Anders H.-J. (2014). Regulated cell death and inflammation: an auto-amplification loop causes organ failure. *Nature Reviews Immunology*.

[6] Hotchkiss R. S., Swanson P. E., Freeman B. D. (1999). Apoptotic cell death in patients with sepsis, shock, and multiple organ dysfunction. *Critical Care Medicine*.

[7] Kidd P. M. (2008). Alzheimer's disease, amnestic mild cognitive impairment, and age-associated memory impairment: Current understanding and progress toward integrative prevention. *Alternative Medicine Review*.

[8] Risner M. E., Saunders A. M., Altman J. F. B. (2006). Efficacy of rosiglitazone in a genetically defined population with mild-to-moderate Alzheimer's disease. *The Pharmacogenomics Journal*.

[9] Stichel C. C., Zhu X. R., Bader V., Linnartz B., Schmidt S., Lübbert H. (2007). Mono- and double-mutant mouse models of Parkinson's disease display severe mitochondrial damage. *Human Molecular Genetics*.

[10] Lin M. T., Beal M. F. (2006). Mitochondrial dysfunction and oxidative stress in neurodegenerative diseases. *Nature*.

[11] Lowell B. B., Shulman G. I. (2005). Mitochondrial dysfunction and type 2 diabetes. *Science*.

[12] Blanche S., Tardieu M., Rustin P. (1999). Persistent mitochondrial dysfunction and perinatal exposure to antiretroviral nucleoside analogues. *The Lancet*.

[13] Chan D. C. (2006). Mitochondria: dynamic organelles in disease, aging, and development. *Cell*.

[14] Schon E. A., Manfredi G. (2003). Neuronal degeneration and mitochondrial dysfunction. *The Journal of Clinical Investigation*.

[15] Kujoth G. C., Hiona A., Pugh T. D. (2005). Medicine: mitochondrial DNA mutations, oxidative stress, and apoptosis in mammalian aging. *Science*.

[16] Yakes F. M., Van Houten B. (1997). Mitochondrial DNA damage is more extensive and persists longer than nuclear DNA damage in human cells following oxidative stress. *Proceedings of the National Acadamy of Sciences of the United States of America*.

[17] Linnane A. W., Marzuki S., Ozawa T., Tanaka M. (1989). Mitochondrial DNA mutations as an important contributor to ageing and degenerative diseases. *The Lancet*.

[18] Sherer T. B., Betarbet R., Greenamyre J. T. (2002). Environment, mitochondria, and Parkinson's disease. *The Neuroscientist*.

[19] Di Monte D. A. (2003). The environment and Parkinson's disease: is the nigrostriatal system preferentially targeted by neurotoxins?. *The Lancet Neurology*.

[20] Rudel R. A., Camann D. E., Spengler J. D., Korn L. R., Brody J. G. (2003). Phthalates, alkylphenols, pesticides, polybrominated diphenyl ethers, and other endocrine-disrupting compounds in indoor air and dust. *Environmental Science & Technology*.

[21] Sonnenschein C., Soto A. M. (1998). An updated review of environmental estrogen and androgen mimics and antagonists. *The Journal of Steroid Biochemistry and Molecular Biology*.

[22] Valavanidis A., Vlahogianni T., Dassenakis M., Scoullos M. (2006). Molecular biomarkers of oxidative stress in aquatic organisms in relation to toxic environmental pollutants. *Ecotoxicology and Environmental Safety*.

[23] Delacroix S., Vogelsang C., Tobiesen A., Liltved H. (2013). Disinfection by-products and ecotoxicity of ballast water after oxidative treatment - Results and experiences from seven years of full-scale testing of ballast water management systems. *Marine Pollution Bulletin*.

[24] Hanson M. L., Sibley P. K., Mabury S. A., Solomon K. R., Muir D. C. (2002). Trichloroacetic acid (TCA) and trifluoroacetic acid (TFA) mixture toxicity to the macrophytes Myriophyllum spicatum and Myriophyllum sibiricum in aquatic microcosms. *Science of the Total Environment*.

[25] Kahrilas G. A., Blotevogel J., Stewart P. S., Borch T. (2015). Biocides in hydraulic fracturing fluids: A critical review of their usage, mobility, degradation, and toxicity. *Environmental Science & Technology*.

[26] Plewa M. J., Kargalioglu Y., Vankerk D., Minear R. A., Wagner E. D. (2000). Development of quantitative comparative cytotoxicity and genotoxicity assays for environmental hazardous chemicals. *Water Science and Technology*.

[27] Plewa M. J., Wagner E. D., Richardson S. D. (2017). TIC-Tox: A preliminary discussion on identifying the forcing agents of DBP-mediated toxicity of disinfected water. *Journal of Environmental Sciences*.

[28] Bove F., Shim Y., Zeitz P. (2002). Drinking water contaminants and adverse pregnancy outcomes: a review. *Environmental Health Perspectives*.

[29] Bull R. J., Reckhow D. A., Li X., Humpage A. R., Joll C., Hrudey S. E. (2011). Potential carcinogenic hazards of non-regulated disinfection by-products: Haloquinones, halo-cyclopentene and cyclohexene derivatives, N-halamines, halonitriles, and heterocyclic amines. *Toxicology*.

[30] Jeong C. H., Wagner E. D., Siebert V. R. (2012). Occurrence and toxicity of disinfection byproducts in European drinking waters in relation with the HIWATE epidemiology study. *Environmental Science and Technology*.

[31] Lynberg M., Nuckols J. R., Langlois P. (2001). Assessing exposure to disinfection by-products in women of reproductive age living in Corpus Christi, Texas and Cobb County, Georgia: Descriptive results and methods. *Environmental Health Perspectives*.

[32] Nieuwenhuijsen M. J., Grellier J., Smith R. (2009). The epidemiology and possible mechanisms of disinfection by-products in drinking water. *Philosophical Transactions of the Royal Society A: Mathematical, Physical & Engineering Sciences*.

[33] Woo Y., Lai D., McLain J. L., Manibusan M. K., Dellarco V. (2002). Use of mechanism-based structure-activity relationships analysis in carcinogenic potential ranking for drinking water disinfection by-products. *Environmental Health Perspectives*.

[34] Choi J., Valentine R. L. (2002). Formation of N-nitrosodimethylamine (NDMA) from reaction of monochloramine: A new disinfection by-product. *Water Research*.

[35] Shah A. D., Mitch W. A. (2012). Halonitroalkanes, halonitriles, haloamides, and N-nitrosamines: A critical review of nitrogenous disinfection byproduct formation pathways. *Environmental Science & Technology*.

[36] Sharma V. K. (2002). Potassium ferrate(VI): an environmentally friendly oxidant. *Advances in Environmental Research*.

[37] Von Gunten U. (2003). Ozonation of drinking water: part II. Disinfection and by-product formation in presence of bromide, iodide or chlorine. *Water Research*.

[38] Hayes J. R., Condie L. W., Borzelleca J. F. (1986). Toxicology of haloacetonitriles. *Environmental Health Perspectives*.

[39] Richardson S. D. (2003). Disinfection by-products and other emerging contaminants in drinking water. *TrAC Trends in Analytical Chemistry*.

[40] Richardson S. D., Plewa M. J., Wagner E. D., Schoeny R., DeMarini D. M. (2007). Occurrence, genotoxicity, and carcinogenicity of regulated and emerging disinfection by-products in drinking water: a review and roadmap for research. *Mutation Research*.

[41] Nilsson R., Tasheva M., Jaeger B. (1993). Why different regulatory decisions when the scientific information base is similar?—Human risk assessment. *Regulatory Toxicology and Pharmacology*.

[42] Damalas C. A., Eleftherohorinos I. G. (2011). Pesticide exposure, safety issues, and risk assessment indicators. *International Journal of Environmental Research and Public Health*.

[43] Kümmerer K. (2009). The presence of pharmaceuticals in the environment due to human use: present knowledge and future challenges. *Journal of Environmental Management*.

[44] Bridges J. (2003). Human health and environmental risk assessment: The need for a more harmonised and integrated approach. *Chemosphere*.

[45] Klaine S. J., Koelmans A. A., Horne N. (2012). Paradigms to assess the environmental impact of manufactured nanomaterials. *Environmental Toxicology and Chemistry*.

[46] Kilkenny C., Browne W., Cuthill I. C., Emerson M., Altman D. G. (2010). Animal research: Reporting in vivo experiments: The ARRIVE guidelines. *The Journal of Gene Medicine*.

[47] Ankley G. T., Bennett R. S., Erickson R. J. (2011). Adverse outcome pathways: a conceptual framework to support ecotoxicology research and risk assessment. *Environmental Toxicology and Chemistry*.

[48] Villeneuve D. L., Crump D., Garcia-Reyero N. (2014). Adverse outcome pathway development II: Best practices. *Toxicological Sciences*.

[49] Villeneuve D. L., Crump D., Garcia-Reyero N. (2014). Adverse outcome pathway (AOP) development I: Strategies and principles. *Toxicological Sciences*.

[50] Becker R. A., Ankley G. T., Edwards S. W. (2015). Increasing scientific confidence in adverse outcome pathways: application of tailored bradford-hill considerations for evaluating weight of evidence. *Regulatory Toxicology and Pharmacology*.

[51] Smyth H. F., Carpenter C. P., Weil C. S., Pozzani U. C., Striegel J. A. (1962). Range-Finding Toxicity Data: List VI. *American Industrial Hygiene Association Journal*.

[52] Kimura E. T., Ebert D. M., Dodge P. W. (1971). Acute toxicity and limits of solvent residue for sixteen organic solvents. *Toxicology and Applied Pharmacology*.

[53] Plewa M. J., Wagner E. D., Muellner M. G., Hsu K. M., Richardson S. D. (2007). Comparative Mammalian Cell Toxicity of N-DBPs and C-DBPs. *Abstracts of Papers of the American Chemical Society*.

[54] Plewa M. J., Muellner M. G., Richardson S. D. (2008). Occurrence, synthesis, and mammalian cell cytotoxicity and genotoxicity of haloacetamides: An emerging class of nitrogenous drinking water disinfection byproducts. *Environmental Science & Technology*.

[55] Saghir S. A., Rozman K. K. (2003). Kinetics of monochloroacetic acid at subtoxic and toxic doses in rats after single oral and dermal administrations. *Toxicological Sciences*.

[56] Bhat H. K., Kanz M. F., Campbell G. A., Ansari G. A. (1991). Ninety day toxicity study of chloroacetic acids in rats. *Fundamental and Applied Toxicology*.

[57] Kitchen K. T., Brown J. L. (1988). Biochemical effects of 3 clorinated phenols in rat liver. *Toxicological & Environmental Chemistry*.

[58] Munson A. E., Sain L. E., Sanders V. M. (1982). Toxicology of organic drinking water contaminants: trichloromethane, bromodichloromethane, dibromochloromethane and tribromomethane. *Environmental Health Perspectives*.

[59] Chu I., Secours V., Marino I., Villeneuve D. C. (1980). Acute toxicity of 4 trihalomethanes in male and female rats. *Toxicology and Applied Pharmacology*.

[60] Kirkinezos I. G., Moraes C. T. (2001). Reactive oxygen species and mitochondrial diseases. *Seminars in Cell & Developmental Biology*.

[61] Lenaz G. (2002). The mitochondrial production of reactive oxygen species: mechanisms and implications in human pathology. *IUBMB Life*.

[62] Carreras M. C., Franco M. C., Peralta J. G., Poderoso J. J. (2004). Nitric oxide, complex I, and the modulation of mitochondrial reactive species in biology and disease. *Molecular Aspects of Medicine*.

[63] Ahmed A. E., Jacob S., Campbell G. A., Harirah H. M., Perez-Polo J. R., Johnson K. M. (2005). Fetal origin of adverse pregnancy outcome: The water disinfectant by-product chloroacetonitrile induces oxidative stress and apoptosis in mouse fetal brain. *Developmental Brain Research*.

[64] Sharma V. K., Dutta P. K., Ray A. K. (2007). Review of kinetics of chemical and photocatalytical oxidation of Arsenic(III) as influenced by pH. *Journal of Environmental Science and Health*.

[65] Ahmed A. E., Jacob S., Nouraldeen A. M. (1999). Chloroacetonitrile (CAN) induces glutathione depletion and 8-hydroxylation of guanine bases in rat gastric mucosa. *Journal of Biochemical and Molecular Toxicology*.

[66] Pals J., Attene-Ramos M. S., Xia M., Wagner E. D., Plewa M. J. (2013). Human cell toxicogenomic analysis linking reactive oxygen species to the toxicity of monohaloacetic acid drinking water disinfection byproducts. *Environmental Science & Technology*.

[67] Li J., Moe B., Vemula S., Wang W., Li X.-F. (2016). Emerging disinfection byproducts, halobenzoquinones: effects of isomeric structure and halogen substitution on cytotoxicity, formation of reactive oxygen species, and genotoxicity. *Environmental Science & Technology*.

[68] Ilett K. F., Reid W. D., Sipes I. G., Krishna G. (1973). Chloroform toxicity in mice: Correlation of renal and hepatic necrosis with covalent binding of metabolites to tissue macromolecules. *Experimental and Molecular Pathology*.

[69] Ekström T., Högberg J. (1980). Chloroform-induced glutathione depletion and toxicity in freshly isolated hepatocytes. *Biochemical Pharmacology*.

[70] Reeve A., Simcox E., Turnbull D. (2014). Ageing and Parkinson's disease: Why is advancing age the biggest risk factor?. *Ageing Research Reviews*.

[71] Meyers D. E., Basha H. I., Koenig M. K. (2013). Mitochondrial cardiomyopathy: Pathophysiology, diagnosis, and management. *Texas Heart Institute Journal*.

[72] Nel A. (2005). Air pollution-related illness: effects of particles. *Science*.

[73] Halliwell B. (1991). Reactive oxygen species in living systems: Source, biochemistry, and role in human disease. *American Journal of Medicine*.

[74] Bagchi D., Bagchi M., Hassoun E. A., Stohs S. J. (1995). In vitro and in vivo generation of reactive oxygen species, DNA damage and lactate dehydrogenase leakage by selected pesticides. *Toxicology*.

[75] Kulms D., Zeise E., Pöppelmann B., Schwarz T. (2002). DNA damage, death receptor activation and reactive oxygen species contribute to ultraviolet radiation-induced apoptosis in an essential and independent way. *Oncogene*.

[76] Sharkey T. D. (2005). Effects of moderate heat stress on photosynthesis: Importance of thylakoid reactions, rubisco deactivation, reactive oxygen species, and thermotolerance provided by isoprene. *Plant, Cell & Environment*.

[77] Apel K., Hirt H. (2004). Reactive oxygen species: metabolism, oxidative stress, and signal transduction. *Annual Review of Plant Biology*.

[78] Bruskov V. I., Malakhova L. V., Masalimov Z. K., Chernikov A. V. (2002). Heat-induced formation of reactive oxygen species and 8-oxoguanine, a biomarker of damage to DNA. *Nucleic Acids Research*.

[79] Carlson C., Hussein S. M., Schrand A. M. (2008). Unique cellular interaction of silver nanoparticles: size-dependent generation of reactive oxygen species. *The Journal of Physical Chemistry B*.

[80] Sayes C. M., Gobin A. M., Ausman K. D., Mendez J., West J. L., Colvin V. L. (2005). Nano-C60 cytotoxicity is due to lipid peroxidation. *Biomaterials*.

[81] Lujan H., Sayes C. M. (2017). Cytotoxicological pathways induced after nanoparticle exposure: Studies of oxidative stress at the 'nano-bio' interface. *Toxicology Research*.

[82] Bender A. (2013). TOM40 mediates mitochondrial dysfunction induced by alpha-synuclein accumulation in Parkinson's disease. *PLoS ONE*.

[83] Olive P. L., Johnston P. J. (1997). DNA damage from oxidants: Influence of lesion complexity and chromatin organization. *Oncology Research : Featuring Preclinical and Clinical Cancer Therapeutics*.

[84] Toyokuni S. (1999). Reactive oxygen species-induced molecular damage and its application in pathology. *Pathology International*.

[85] Launay N., Ruiz M., Fourcade S. (2013). Oxidative stress regulates the ubiquitin-proteasome system and immunoproteasome functioning in a mouse model of X-adrenoleukodystrophy. *Brain*.

[86] Chandel N. S., McClintock D. S., Feliciano C. E. (2000). Reactive oxygen species generated at mitochondrial Complex III stabilize hypoxia-inducible factor-1*α* during hypoxia: a mechanism of O_2_ sensing. *The Journal of Biological Chemistry*.

[87] Kim S.-J., Cheresh P., Jablonski R. P., Williams D. B., Kamp D. W. (2015). The role of mitochondrial DNA in mediating alveolar epithelial cell apoptosis and pulmonary fibrosis. *International Journal of Molecular Sciences*.

[88] Li L., Nadanaciva S., Berger Z. (2013). Human A53T *α*-synuclein causes reversible deficits in mitochondrial function and dynamics in primary mouse cortical neurons. *PLoS ONE*.

[89] Mailloux R. J., McBride S. L., Harper M.-E. (2013). Unearthing the secrets of mitochondrial ROS and glutathione in bioenergetics. *Trends in Biochemical Sciences*.

[90] Vaziri N. D., Wang X. Q., Oveisi F., Rad B. (2000). Induction of oxidative stress by glutathione depletion causes severe hypertension in normal rats. *Hypertension*.

[91] Övey İ. S., Naziroğlu M. (2015). Homocysteine and cytosolic GSH depletion induce apoptosis and oxidative toxicity through cytosolic calcium overload in the hippocampus of aged mice: involvement of TRPM2 and TRPV1 channels. *Neuroscience*.

[92] Lu C., Armstrong J. S. (2007). Role of calcium and cyclophilin D in the regulation of mitochondrial permeabilization induced by glutathione depletion. *Biochemical and Biophysical Research Communications*.

[93] Baumgartner H. K., Gerasimenko J. V., Thorne C. (2009). Calcium elevation in mitochondria is the main Ca2+ requirement for mitochondrial permeability transition pore (mPTP) opening. *The Journal of Biological Chemistry*.

[94] Bernardi P. (1992). Modulation of the mitochondrial cyclosporin A-sensitive permeability transition pore by the proton electrochemical gradient: evidence that the pore can be opened by membrane depolarization. *The Journal of Biological Chemistry*.

[95] Almeida A., Heales S. J. R., Bolaños J. P., Medina J. M. (1998). Glutamate neurotoxicity is associated with nitric oxide-mediated mitochondrial dysfunction and glutathione depletion. *Brain Research*.

[96] Bruce J. I. E. (2010). Plasma membrane calcium pump regulation by metabolic stress. *World Journal of Biological Chemistry*.

[97] Santos J. H., Hunakova L., Chen Y., Bortner C., Van Houten B. (2003). Cell sorting experiments link persistent mitochondrial DNA damage with loss of mitochondrial membrane potential and apoptotic cell death. *The Journal of Biological Chemistry*.

[98] Ehlers R. A., Hernandez A., Bloemendal L. S., Ethridge R. T., Farrow B., Evers B. M. Mitochondrial DNA damage and altered membrane potential (Delta Psi) in pancreatic acinar cells induced by reactive oxygen species. *Surgery*.

[99] Du Y., Ma Z., Lin S. (2001). Minocycline prevents nigrostriatal dopaminergic neurodegeneration in the MPTP model of Parkinson's disease. *Proceedings of the National Acadamy of Sciences of the United States of America*.

[100] Hernandez-Baltazar D., Mendoza-Garrido M. E., Martinez-Fong D. (2013). Activation of GSK-3*β* and caspase-3 occurs in nigral dopamine neurons during the development of apoptosis activated by a striatal injection of 6-hydroxydopamine. *PLos One*.

[101] Ares-Santos S., Granado N., Espadas I., Martinez-Murillo R., Moratalla R. (2014). Methamphetamine causes degeneration of dopamine cell bodies and terminals of the nigrostriatal pathway evidenced by silver staining. *Neuropsychopharmacology*.

[102] Crouser E. D. (2004). Mitochondrial dysfunction in septic shock and multiple organ dysfunction syndrome. *Mitochondrion*.

